# Serum E-selectin concentration is associated with risk of metabolic syndrome in females

**DOI:** 10.1371/journal.pone.0222815

**Published:** 2019-09-24

**Authors:** Chien-Hsing Lee, Feng-Chih Kuo, Wen-Hao Tang, Chieh-Hua Lu, Sheng-Chiang Su, Jhih-Syuan Liu, Chang-Hsun Hsieh, Yi-Jen Hung, Fu-Huang Lin

**Affiliations:** 1 Division of Endocrinology and Metabolism, Department of Internal Medicine, Tri-Service General Hospital, National Defense Medical Center, Taipei, Taiwan, ROC; 2 Division of Endocrinology and Metabolism, Department of Internal Medicine, Tri-Service General Hospital, Song Shan Branch, Taipei, Taiwan, ROC; 3 School of Public Health, National Defense Medical Center, Taipei, Taiwan, ROC; International University of Health and Welfare, School of Medicine, JAPAN

## Abstract

**Objectives:**

Traits of metabolic syndrome (MetS) and biomarkers of inflammation and endothelial dysfunction were examined. We investigated the differences of various biomarkers among individuals with or without Mets in a gender-specific manner. The gender-specific associations between E-selectin and MetS were further evaluated.

**Methods:**

A total of 205 patients were recruited from the outpatient clinics of Tri-Service General Hospital, Taipei, Taiwan. Inclusion criteria were age between 20–75 years and BMI < 35 kg/m^2^. Demographic, anthropometric and MetS index data were compared between genders. Markers of inflammation and endothelial dysfunction were compared between individuals with or without MetS by gender.

**Results:**

Age-adjusted E-selectin values showed significant positive correlations with BMI, waist-hip ratio, fasting plasma glucose, systolic and diastolic blood pressure, triglycerides, TNF-α, hsCRP and ICAM-1, and inverse correlation with HDL cholesterol. E-selectin levels were positively correlated with numbers of MetS components in females (P < 0.001) but not in males (P = 0.125).

**Conclusions:**

Increased E-selectin levels are significantly associated with increased MetS risk in females, but not in males.

## Introduction

Metabolic syndrome (MetS) is a cluster of conditions, including hypertension, hyperglycemia, abdominal obesity and hyperlipidemia, that increase risk for cardiovascular disease, stroke and diabetes [1]. The incidence of MetS is increasing and is becoming a challenge to public health worldwide. Prevalence of MetS in the Mediterranean region of Turkey was 34% in the overall population and 43.2% in obese patients aged 60–69 years, and even higher (56.8%) among those with abdominal obesity [2]. Prevalence of MetS in urban Pakistan was 34.8% according to the International Diabetes Federation (IDF) definition and 49% according to the modified Adult Treatment Panel III (ADPIII) criteria, which includes modified cut-offs for waist circumference and body mass index (BMI) for Asian populations [3]. In Asian countries, even though the prevalence of obesity is relatively lower compared to western countries, comparative studies have shown that obesity-related metabolic cardiovascular risks at given BMIs may be higher in South and East Asian populations [4]. South Asian populations have higher atherogenic dyslipidemia, glucose intolerance, thrombotic tendency, subclinical inflammation and endothelial dysfunction than Caucasians, and may display cardiovascular disorders even at lower levels of adiposity and abdominal obesity [5].

In Taiwan, the age-standardized prevalence of MetS was 15.7% (18.3% in men, 13.6% in women) by the modified ATP III criteria, which is slightly lower than other Asian countries. However, the prevalence increases significantly with age, particularly in post-menopausal women, and reaches its peak in the 7th decade of life (32.9% in men, 41.4% in women) [6]. For elderly individuals (≥65 y/o) in Taiwan, the MetS risk and prevalence is relatively high even under non-obese status [7]. Also, the risk for having MetS is higher among elder men (age 65–80 years) with high normotension [8]. Besides, in some populations, a gender-specific difference in the prevalence of MetS has been observed, with generally higher prevalence found in women [9, 10]. Therefore, multiple factors including age, gender and race need to be taken into account when evaluating the risk and prevalence of MetS.

Associations between MetS and biomarkers of inflammation and endothelial dysfunction have been previously established. Measuring the serum concentration of these markers in patients could potentially stratify the risk of individuals for possessing the characteristic MetS components and associated diseases such as cardiovascular disease and diabetes. Inflammatory cytokines, tumor necrosis factor-alpha (TNF-α) and interleukin-6 (IL-6) are all recognized as components of the inflammatory mechanisms accompanying the status of obesity and MetS [11, 12]. Adhesion molecules such as selectins, intercellular vascular adhesion molecule-1 (ICAM-1) and vascular cell adhesion moloecule-1 (VCAM-1) are expressed both in the endothelium and certain types of leukocytes, allowing them to recruit circulating leukocytes into the endothelium to initiate atherogenesis [13]. As such, E-selectin is an endothelial adhesion molecule known to be integrally involved in the development of atherosclerotic plaque by promoting the adhesion of leukocytes to the endothelial wall [14]. Levels of E-selectin are also increased in obesity, specifically in association with increased visceral adiposity and increased markers of TNF-α activation [15]. In our previous study [16], which investigated the association of plasma protein growth arrest-specific 6 (Gas6) levels with altered glucose tolerance, inflammation and endothelial dysfunction, we noted that E-selectin concentration was associated with established diabetes risk factors, suggesting that its association with MetS should be further evaluated.

Although some previous studies may have accounted for differences in MetS components between males and females, no studies have focused on comparing the levels of established biomarkers for MetS in a gender-specific manner. E-selectin has been acknowledged to have a potential relationship with MetS, but to the best of our knowledge, no definitive correlation has been directly examined. Therefore, the purpose of the present study was to investigate the potential association of E-selectin, as a marker of endothelial dysfunction, with the traits of MetS. At the same time, we also examined whether the levels of E-selectin and various biomarkers are different among individuals with or without MetS in a gender-specific manner.

## Materials and methods

### Study population

A total of 205 subjects were recruited for this cross-sectional study from the outpatient clinics of Tri-Service General Hospital, Taipei, Taiwan. The inclusion criteria were: age 20–75 years, BMI <35 kg/m^2^; absence of infection within the previous weeks; not receiving oral anticoagulants or antidiabetes therapy (e.g., oral hypoglycemic agents, insulin, and glucagon-like peptide 1); and no history of malignancy. Women who were pregnant or breast feeding; patients with impaired renal function (serum creatinine ≥132.6 mol/l); patients with abnormal serum aspartate aminotransferase or alanine aminotransferase (2.5 times above the upper normal ranges); patients with acute or chronic pancreatitis; patients with a history of cerebrovascular accident, myocardial infarction, or heart failure; patients with autoimmune disorders or psychiatric diseases, including mood disorders and alcoholism; and patients taking concomitant drugs such as ß-blockers, diuretics, cholestyramine, or systemic steroids were excluded. Of these, 205 patients who met the inclusion criteria were enrolled in this study, including 88 males and 117 females with a median age of 56 years. The study protocol was reviewed and approved by the institutional review board of Tri-Service General Hospital. All included patients provided signed informed consent to participate in the study. Subjects were assured that their data would be used only for the purpose of this study and that they would remain anonymous in any published reports of the study.

### Main outcome measures

The demographic, anthropometric and clinical data of all patients were collected for evaluation in two groups classified by gender and by MetS vs. non-MetS. After 10 hour of fastening, blood samples were obtained to determine plasma glucose, and lipid profiles. Plasma circulating high sensitive C-reactive protein (hsCRP), tumor necrosis factor-a (TNF-a) and interleukin-6 (IL-6) levels, E-selectin, intercellular adhesion molecule 1 (ICAM-1) and vascular cell adhesion molecule-1 (VCAM-1) were subsequently measured. Serum triglyceride was measured using the dry, multilayer analytical slide method in the Fuji Dri-Chem 3000 analyzer (Fuji Photo Film Corporation, Tokyo, Japan). The intra-assay and inter-assay coefficients of variance (CV) for triglyceride were 0.8% and 2.5%, respectively. Serum levels of high-density lipoprotein cholesterol (HDL-C) were determined by an enzymatic cholesterol assay method after dextran sulfate precipitation. The intra-assay and inter-assay CV for HDL-C were 1.1% and 1.7%, respectively. Plasma glucose concentrations were determined by the glucose oxidase method on a Beckman Glucose Analyzer II (Beckman Instruments, Fullerton, CA, USA). The intra-assay and inter-assay CV for glucose were 0.6% and 1.5%, respectively. Plasma hsCRP levels were measured using the Tina-quant (Latex) high sensitivity assay (Roche, Mannheim, Germany). The intra-assay and inter-assay CV for hsCRP were 3.7% and 4.9%, respectively. Serum IL-6 concentrations were determined by a human high sensitivity enzyme-linked-immunosorbent assay (ELISA) (Besancon Cedex, France). The intra-assay and inter-assay CV for IL-6 were 1.5% and 5.3%, respectively. Serum TNF-a was measured with the BiotrakTM high sensitivity human ELISA kit from Amersham Biosciences (Buckinghamshire, UK). The intra-assay and inter-assay CV for TNF-a were 3.5% and 5.3%, respectively. Levels of E-selectin, ICAM-1, and VCAM-1 were measured by commercial ELISA (R&D Systems, Minneapolis, USA). The intra-assay and inter-assay CV for E-selectin were 4.5% and 6.2%; ICAM-1 were 3.5% and 7.1%; and VCAM-1 were 5.0% and 8.7%, respectively. All of the concentrations of the above biochemical variables were determined in duplicate and the values of the two samples were averaged.

### Taiwan criteria for defining MetS

For the purpose of this study, the criteria used to define MetS in study subjects was modified from the accepted WHO definition to accommodate the generally smaller stature (e.g., height, weight, waist circumference and BMI) of Taiwanese adults compared to Western counterparts. According to the modified National Cholesterol Education Program Expert Panel Adult Treatment Panel III (NCEP ATP III) criteria [17], patients who have at least 3 of the following indicators are considered to have MetS. (1) Central obesity—defined as waist circumference (WC) ≥ 90 cm for men and ≥ 80 cm for women; (2) Elevated triglyceride level ≥ 150 mg/dl or specific treatment for this lipid abnormality; (3) Reduced high density lipoprotein cholesterol (HDL) level < 40 mg/dL (1.03 mmol/L) in males and < 50 mg/dL (1.29 mmol/L) in females,; (4) Elevated blood pressure (BP)—systolic BP (SBP) ≥ 130 mmHg or diastolic BP (DBP) ≥ 85 mmHg or treatment of previously diagnosed hypertension; (5) Elevated fasting plasma glucose (FPG) ≥ 100 mg/dL (5.6 mmol/L), or previously diagnosed type 2 diabetes mellitus. If the level of FPG is over 100 mg/dL (> 5.6 mmol/L), oral glucose tolerance test (OGTT) is strongly recommended but is not necessary to define presence of MetS.

### Statistical analysis

Continuous variables are summarized as mean ± standard deviation (SD) or median with interquartile range (IQR) depending on normality of data distribution. Categorical variables are summarized as frequencies and percentages. Demographic characteristics and biochemical data were compared between two groups (male vs. female, MS vs. non-MS) using independent t-test or Wilcoxon rank sum test for continuous variables, and Chi-square test for categorical variables, as appropriate. Correlations between E-selectin and other biochemical variables were measured using Spearman’s correlation coefficient and partial correlation analysis after adjusting for age. Spearman’s correlation coefficient was also used to investigate the linear trend of E-selectin levels across numbers of MetS components. The receiver operating characteristic (ROC) curves were used to examine the diagnostic performance characteristics of E-selectin and inflammatory markers (TNF-α, IL-6, hsCRP) as well as other endothelial dysfunction markers (ICAM-1, VCAM-1) to distinguish patients with MetS from those who were non-MetS; the point estimate with 95% confidence interval (CI) of the area under ROC curve (AUC) was provided as an index to compare global test performance. The optimal cutoff values were determined by maximizing the sum of sensitivity and specificity. The ROC contrast tests were computed to compare the different AUC values. Subgroup analysis was performed according to gender. Statistical analyses were performed using SAS software version 9.2 (SAS Institute, Cary, NC). A two-tailed P < 0.05 represented statistical significance.

## Results

### Baseline characteristics and index of MetS between males and females

A total of 205 subjects were enrolled in this study, 88 males and 117 females with median age of 56 years (range: 20 to 75 years). Among all patients, 84 (41.0%) were identified as having MetS according to the Taiwan MetS criteria (see Methods above). Comparisons of baseline demographic characteristics and index of MetS between males and females are shown in Table 1. More males than females smoked (44.3% vs. 4.3%; P < 0.001) and consumed alcohol (30.7% vs. 6.8%; P < 0.001). Regarding the index of MetS, males had significantly higher fasting plasma glucose (P = 0.026), diastolic blood pressure (P = 0.015), waist circumference (P < 0.001), and waist-to-hip ratio (P < 0.001), but lower HDL cholesterol (P < 0.001) compared to females. However, the prevalence of MetS did not differ between males and females (42.1% vs. 40.2%; P = 0.787). (Table 1)

**Table 1:** Comparison of baseline characteristics (age, BMI, smoking, and alcohol consumption) and index of MS (fasting blood glucose, blood pressure, TG, HDL-C, waist circumference) between males and females.

	Total (N = 205)	Male (N = 88)	Female (N = 117)	P-value
*Demographic and clinical characteristics*				
Age (year) ^2^	56.0 (48.0, 63.0)^a^	55.0 (46.0, 61.0)^b^	58.0 (51.0, 63.0)	0.056
BMI (kg/m^2^) ^2^	24.7 (22.2, 27.3)	24.6 (22.5, 26.7)	24.7 (22.0, 27.4)	0.853
Smoking^3^	44 (21.5)	39 (44.3)	5 (4.3)	**< 0.001**
Alcohol consumption^3^	35 (17.1)	27 (30.7)	8 (6.8)	**< 0.001**
Family history of diabetes^3^	82 (40.0)	39 (44.3)	43 (36.8)	0.274
Metabolic syndrome^3^	84 (41.0)	37 (42.1)	47 (40.2)	0.787
*Index of metabolic syndrome*	* *			
Fasting plasma glucose (mmol/l) ^2^	98.0 (88.6, 113.0)	101.5 (90.9, 117.0)	96.3 (86.2, 106.0)	**0.026**
Systolic blood pressure (mmHg) ^2^	122.0 (112.0, 132.0)	122.0 (110.0, 130.0)	122.0 (114.0, 136.0)	0.534
Diastolic blood pressure (mmHg) ^1^	79.2 ± 9.5	81.0 ± 9.2	77.8 ± 9.6	**0.015**
Triglycerides (mmol/l) ^2^	127.0 (87.0, 169.0)	132.5 (97.5, 179.5)	116.0 (84.0, 167.0)	0.207
HDL cholesterol (mmol/l) ^2^	45.4 (38.6, 58.0)	42.0 (35.0, 47.0)	54.0 (41.8, 65.0)	**< 0.001**
Waist circumference (cm) ^1^	84.3 ± 10.6	89.0 ± 9.6	80.7 ± 9.8	**< 0.001**
Waist-to-hip ratio^2^	0.88 (0.83, 0.92)	0.92 (0.88, 0.94)	0.84 (0.81, 0.88)	**< 0.001**

^1^ Normally distributed continuous data are presented as mean ± standard deviation (SD).

^2^ Non-normally distributed continuous data are presented as median (interquartile range, IQR).

^3^ Categorical data are expressed as counts (%)

^a^ Due to one subject with missing data of age, N = 204

^b^ Due to one subject with missing data of age, N = 87

### Inflammatory markers and endothelial dysfunction markers in MetS and non-MetS patients

Among males, patients with MetS had significantly higher levels of IL-6 [median (IQR): 2.07 (1.25, 4.17) pg/ml vs. 1.22 (0.73, 2.66) pg/ml; P = 0.023] and hsCRP [median (IQR): 0.49 (0.40, 0.98) mg/dl vs. 0.38 (0.27, 0.75) mg/dl; P = 0.038] than subjects without MetS (Table 2). The level of E-selectin was higher in the MetS group than in the non-MetS group [median (IQR): 55.9 (37.7, 80.9) ng/ml vs. 45.8 (33.7, 66.0) ng/ml; P = 0.075], but the difference was not significant.

**Table 2:** Inflammatory markers (TNF-α, IL-6, hsCRP) and endothelial dysfunction markers (E-selectin, ICAM-1, VCAM-1) between MetS and non-MetS patients by gender.

	Male (N = 88)		Female (N = 117)			
	non-MS (N = 51)	MS (N = 37)	P-value	non-MS (N = 70)	MS (N = 47)	P-value
*Inflammatory markers*						
TNF-α(ng/ml)	2.92 (1.90, 3.68)	3.34 (1.70, 4.51)	0.432	2.92 (1.67, 3.58)	2.64 (1.23, 4.23)	0.956
IL-6 (pg/ml)	1.22 (0.73, 2.66)	2.07 (1.25, 4.17)	**0.023**	1.07 (0.50, 2.22)	1.88 (0.88, 2.79)	**0.023**
hsCRP (mg/dl)	0.38 (0.27, 0.75)	0.49 (0.40, 0.98)	**0.038**	0.47 (0.27, 1.09)	1.03 (0.51, 2.19)	**< 0.001**
*Endothelial dysfunction markers*						
E-selectin (ng/ml)	45.8 (33.7, 66.0)	55.9 (37.7, 80.9)	0.075	40.0 (30.0, 51.4)	56.8 (31.3, 81.0)	**< 0.001**
VCAM-1 (ng/ml)	457.0 (307.6, 669.6)	546.1 (348.8, 795.8)	0.157	366.3 (239.8, 570.2)	544.3 (287.7, 846.0)	**0.041**
ICAM-1 (ng/ml)	252.3 (208.8, 320.9)	269.0 (207.4, 326.5)	0.636	252.0 (187.9, 295.2)	253.3 (205.6, 321.3)	0.305

Among females, patients with MetS also had significantly higher levels of IL-6 [median (IQR): 1.88 (0.88, 2.79) pg/ml vs. 1.07 (0.50, 2.22) pg/ml; P = 0.023] and hsCRP [median (IQR): 1.03 (0.51, 2.19) mg/dl vs. 0.47 (0.27, 1.09) mg/dl; P < 0.001] than subjects without MetS (Table 2). MetS patients also had significantly higher E-selectin [median (IQR): 56.8 (31.3, 81.0) ng/ml vs. 40.0 (30.0, 51.4) ng/ml; P < 0.001] and VCAM-1 [median (IQR): 544.3 (287.7, 846.0) ng/ml vs. 366.3 (239.8, 570.2) ng/ml; P = 0.041] compared to those without MetS (Table 2).

### Age-adjusted correlation between E-selectin and other biochemical variables

E-selectin values in all patients correlated significantly and positively with BMI, waist-to-hip ratio, FPG, SBP, DBP, triglycerides, TNF-α, hsCRP and ICAM-1, but correlated inversely with HDL cholesterol after adjusting for age (Table 3). In females, results of these partial but significant correlations between E-selectin and other variables were maintained as that for all patients, but in males, the E-selectin value correlated significantly with only BMI, waist-to-hip ratio, and triglycerides as well as ICAM-1 after adjusting for age. (Table 3)

**Table 3:** Age-adjusted Spearman partial correlation coefficients between E-selectin concentration and anthropometric (BMI, waist-to-hip ratio) and biochemical variables by gender.

	All (N = 204) ^a^		Male (N = 87) ^b^		Female (N = 117)	
	r_s_	P-value	r_s_	P-value	r_s_	P-value
BMI (kg/m^2^)	0.260	**< 0.001**	0.359	**0.001**	0.193	**0.038**
Waist-to-hip ratio	0.322	**< 0.001**	0.290	**0.007**	0.301	**0.001**
Fasting plasma glucose (mmol/l)	0.257	**< 0.001**	0.094	0.391	0.319	**0.001**
Systolic blood pressure (mmHg)	0.175	**0.012**	0.102	0.349	0.220	**0.018**
Diastolic blood pressure (mmHg)	0.198	**0.005**	0.148	0.173	0.196	**0.035**
Triglycerides (mmol/l)	0.348	**< 0.001**	0.362	**0.001**	0.307	**0.001**
HDL cholesterol (mmol/l)	-0.184	**0.009**	-0.066	0.546	-0.217	**0.019**
TNF-α (ng/ml)	0.218	**0.002**	0.189	0.082	0.273	**0.003**
IL-6 (pg/ml)	0.108	0.127	0.162	0.136	0.031	0.743
hsCRP (mg/dl)	0.174	**0.013**	0.194	0.073	0.205	**0.027**
VCAM-1 (ng/ml)	-0.003	0.961	0.094	0.392	-0.112	0.232
ICAM-1 (ng/ml)	0.273	**< 0.001**	0.267	**0.013**	0.253	**0.006**

^a^ Due to one subject with missing data of age, N = 204

^b^ Due to one subject with missing data of age, N = 87

### Linear trend of E-selectin levels with number of MetS components

**Fig 1:**
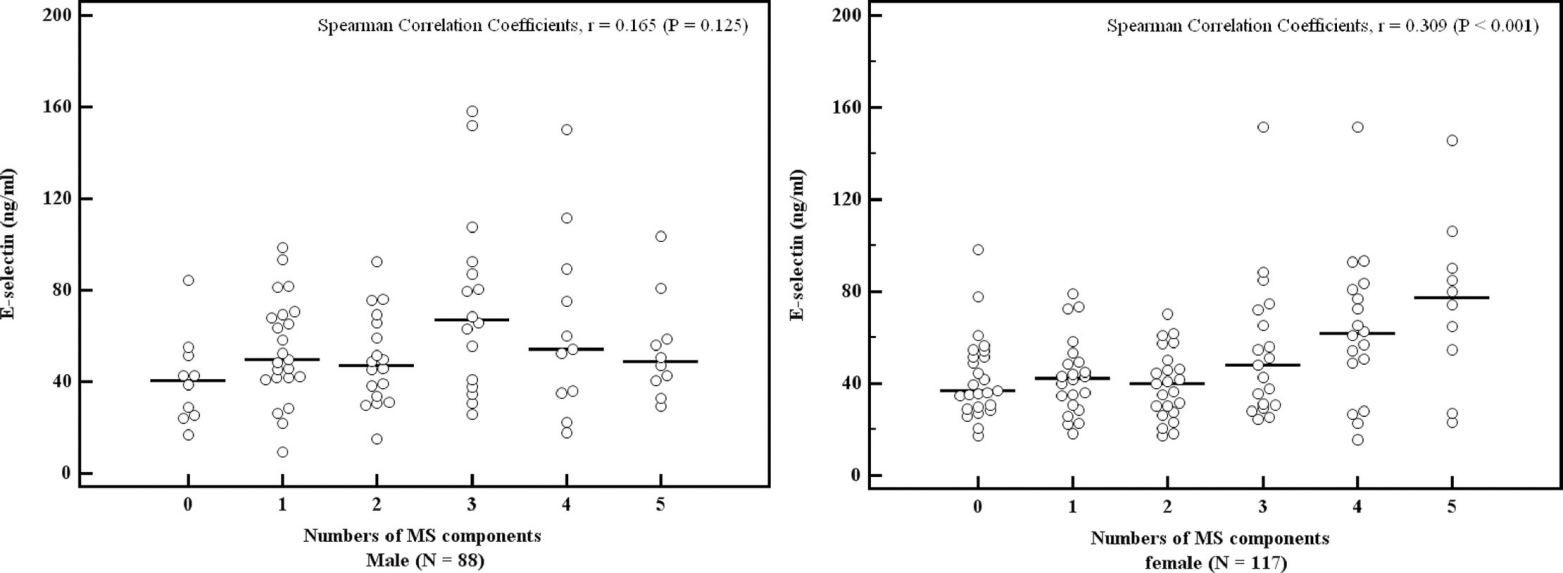
The level of plasma E-selectin concentration across different numbers of MS components (0 to 5 components) by gender. The circles represent individual values of each patient, and the lines represent median values in each category.

Fig 1 presents the distribution of E-selectin levels across numbers of MetS components in males and females. No significant associations were found between E-selectin levels and the number of MetS components among males (P = 0.125), while elevated E-selectin levels in females were significantly associated with the number of MetS components (P < 0.001).

### ROC curve analysis using inflammatory markers and endothelial dysfunction markers to detect MetS status

**Fig 2:**
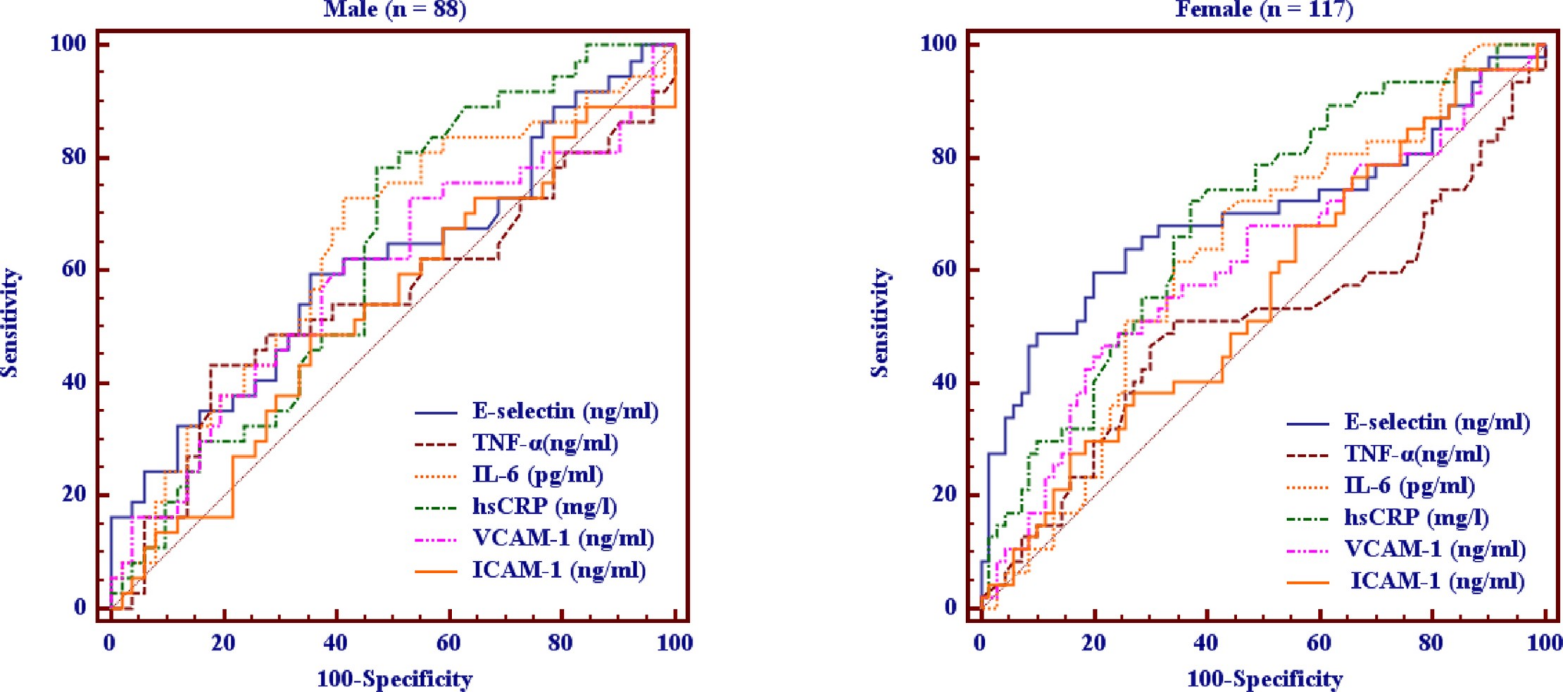
ROC curve analysis of values for inflammatory markers (TNF-α, IL-6, hsCRP) and endothelial dysfunction markers (E-selectin, ICAM-1, VCAM-1) in the detection of MetS status, as shown by gender.

ROC curves using TNF-α, IL-6, hsCRP, E-selectin, ICAM-1, and VCAM-1 to differentiate patients with MetS from those without MetS are shown in Fig 2 by gender. The AUC of E-selectin (0.612, 95% CI = 0.502 to 0.714) did not differ from those of other biomarkers among males. However, the AUC of E-selectin (0.695, 95% CI = 0.603 to 0.776) was significantly higher than that of TNF-α (0.503, 95% CI = 0.409 to 0.597) and ICAM-1 (0.556, 95% CI = 0.461 to 0.648) among females (P = 0.032 and 0.033, respectively).

## Discussion

This is the first study to demonstrate a positive association between E-selectin and MetS only in females, which highlights E-selectin has differential association with MetS across genders. The prevalence of MetS did not differ between males and females, but males had significantly higher MetS index factors than females, including FPG, DBP, waist circumference, and waist-to-hip ratio, but lower HDL cholesterol. Among the markers of inflammation and endothelial dysfunction in patients with and without MetS, males with MetS had significantly higher levels of IL-6 and hsCRP than the non-MetS group, and also had higher E-selectin levels but without significance. Female patients with MetS also had significantly higher levels of IL-6 and hsCRP than non-MetS patients and significantly higher E-selectin and VCAM-1 than the non-MetS group. E-selectin values also showed significant, positive correlations with BMI, waist-to-hip ratio, FPG, SBP, DPB, triglycerides, TNF-α, hsCRP and ICAM-1, but inverse correlations with HDL cholesterol, which were all preserved in the female-predominant pattern. In the gender-specific analysis, we evaluated E-selectin levels across the number of MetS components and elevated E-selectin levels were only significantly correlated with the number of MetS components in female group but not in male group.

There were several studies have described the increased levels of circulating soluble E-selectin in patients with diabetes, obesity, and coronary artery disease, and soluble VCAM-1 and/or soluble ICAM-1 are increased in patients with metabolic syndrome who involve both male and female [18–23]. However, all of the above previous studies didn’t provide the detail data and analysis that especially in female and in male only. In our study, E-selectin values revealed significant, positive correlations with BMI, waist-to-hip ratio, FPG, SBP, DPB, triglycerides, TNF-α, hsCRP and ICAM-1, but inverse correlations with HDL cholesterol, which are were all preserved in the female-predominant pattern. We could explain this gender-specificity by hormonal interactions. The role of selectins in female reproduction has already been established, as they are involved in the regulation of the ovarian function, menopause and the pathogenesis of preeclampsia [24]. E-selectin can be detected in endometritis cases, but also in follicular aspirates during IVF in human ovarian micro-vascular endothelium [25, 26]. Its levels have been correlated with the clinical presentation and biological aspects of ovarian hyperstimulation syndrome (OHSS) [27], and the differentiated hormonal states induced by the administration of GnRH agonists [26] or oral estrogens [28]. Indeed, Caulin-Glaser et al., in their study on subjects with coronary artery disease, observed a statistically significant increase in soluble E-selectin levels in men and postmenopausal women not receiving estrogen replacement therapy compared with women receiving ERT [29]. On the other hand, study by Elhadd et al. investigating levels of CAMs in three different phases of the menstrual cycle in young healthy normal cycling women reported that changes in estrogen levels during the menstrual cycle can significantly alter the levels of E-selectin, but not ICAM-1 [30]. Previous studies have reported an increase of CAMs in postmenopausal women, when the female sex hormones decline, while the hormonal replacement therapy was successful in decreasing levels of soluble CAMs. Piercy et al. demonstrated that combined estrogen and progesterone exposure significantly decreased the expression of VCAM-1 and ICAM-1 in cell cultures of normal human female iliac artery stimulated endothelial cells [31]. Although we didn’t record the prevalence of menses and measure the hormonal status, sex hormones may affect the association between serum E-selectin and metabolic syndrome in females in our study. Furthermore, the different criteria of metabolic syndrome in different gender groups may also influence the different association between gender groups and metabolic syndrome in this study.

Previous studies have investigated E-selectin in association with diabetes and hypertension [13, 19, 32], and with malignancies [33], but not specifically evaluated its association with MetS. Cominacini et al. (1995) showed that E-selectin plasma concentration was positively correlated with the levels of glycated hemoglobin in diabetic patients, which suggests that the soluble adhesion molecules, especially E-selectin, may be related to metabolic control in diabetic patients [19]. Also, the patients with hyperlipoproteinemia have increased E-selectin concentration compared to the healthy controls, which indicates that increasing cholesterol levels were in some way related to endothelial cell stimulation [19]. The previous report showed the levels of soluble E-selectin, soluble ICAM-1, and soluble VCAM-1 in the serum of patients with hypertriglyceridemia were increased, and multivariable analyses revealed that this increase was independent of other risk factors [32]. Calabresi et al have been described that high levels of soluble E-selectin in subjects with low concentration of HDL in both hyper and normolipidemic patients, and after treatment with fenofibrate, HDL-cholesterol raised and soluble E-selectin was decreased [33]. This is compatible with the in vitro demonstration of a reduced E-selectin expression in response to TNF in endothelial cells that had been pre-incubated with HDL [34]. Furthermore, a group of patients with elevated triglyceride and reduced HDL levels, showed high levels of soluble E-selectin and a significant reduction of soluble E-selectin with an increase in HDL levels after 6 months of treatment, even after adjustment for diabetes, clinical atherosclerosis, hypertension and smoking [32]. These results support previous in vitro data suggesting that disorders of triglyceride and HDL metabolism may promote atherogenesis and vascular cell activation through effects on E-selectin and CAMs. In addition, the combined abnormalities of high triglyceride/low HDL and abnormalities of glucose metabolism, which are part of the insulin resistance syndrome, may synergistically increase the level of E-selectin and CAMs expression.

Subsequently, Matsumoto et al. further disclosed that, in type 2 diabetic patients, BMI and total fat mass are significantly correlated with the concentration of soluble E-selectin. The authors propose that obesity may induce shedding of cell surface E-selectin in the activated endothelium contributing to the increase in soluble E-selectin levels [19]. De Caterina et al. revealed that patients with essential hypertension, plasma levels of soluble E-selectin are higher than in normotensive controls and mostly related to structural vascular changes [35]. The increased hemodynamic stress on the endothelium in hypertension provided a possible mechanism for raised endothelial markers including of soluble E-selectin and von Willebrand factor etc. are reflective of a damaged endothelium that then contributes to hypertension by failing to correctly perform its duties in regulating vascular tone [36]. Furthermore, Wang et al. investigated the association between genetic variants in the E-selectin gene and essential hypertension in Han, Kazakh and Uygur populations in China. They found an association of E-selectin gene variants with essential hypertension in these populations [37]. These reports suggest that E-selectin has an important role in the development of hypertension through causing the impairment of endothelial function. In line with our study, we found E-selectin values in all patients correlated significantly and positively with BMI, waist-to-hip ratio, FPG, SBP, DPB and triglyceride levels, and correlated inversely with HDL cholesterol. Besides, we also observed the positive correlation between fasting glucose and E-selectin concentration. These results point out that soluble E-selectin expression was elevated by obesity-related dyslipidemia and hyperglycemia, that is, possessing at least two components of MetS.

Other adhesion molecules, ICAM-1 and VCAM-1, were also examined in the Matsumoto study, but did not correlate with BMI [19]. In our study, we reveals compatible findings in that, compared to ICAM-1 and VCAM-1, E-selectin is more influenced by obesity. However, in terms of the major adverse cardiovascular events, VCAM-1 concentration is significantly associated with cardiovascular mortality in type 2 diabetes patients [38], and circulating levels of soluble VCAM-1, ICAM-1 are shown to be elevated in diabetic patients with stroke [39]. Moreover, soluble adhesion molecules as indicators of endothelial activation and inflammation are further supported by the consistent results that levels of soluble ICAM-1, VCAM-1 and E-selectin are all significantly higher in patients with angiographically proven slower coronary flow than normal subjects [40]. Notably, amelioration of oxidative stress either via anti-hyperglycemic therapy or simvastatin treatment could also decreases the circulating levels of the adhesion molecules including ICAM-1, VCAM-1 and E-selectin [41, 42]. Combing these studies, it is likely that soluble adhesion molecules, such as E-selectin could be an indicator for the vascular microenvironment such as blood flow, oxidative stress, hyperglycemia and dyslipidemia, particularly in the individuals with obesity-related MetS.

Among the few studies that have looked at the correlation between E-selectin and inflammatory markers in patients with MetS, Trøseid et al. showed that E-selectin levels responded to changes in glycemic control and BMI, but not to changes in levels of TNF-α [43]. Another recent study showed that components of the IL-6 trans-signaling system were significantly elevated in more than 200 subjects with MetS and positively correlated with markers of endothelial dysfunction and arterial stiffness, such as E-selectin, ICAM-1 and VCAM-1 [44]. In contrast to the previous research, we found the levels of E-selectin correlated positively with TNF-α and hsCRP but not with IL-6. Clearly, more studies are still required to unravel the roles and complex interactions between inflammation and endothelial dysfunction biomarkers during the pathophysiological process of atherogenesis among patients with MetS.

### Limitations

The present study has certain limitations, primarily that it was conducted in only one center in one regional location, which limits generalizability to the greater population in Taiwan and precludes estimates of incidence. Also, the subjects were originally recruited for another study prior to 2010 and all the subjects in the present study were therefore lost to follow-up. This limits the conclusions of the study to only to associations between E-selectin and the presence of MetS, but not to the prediction of MetS. Future multi-center prospective studies with a larger sample and longer follow-up are needed to confirm results of the present study regarding the association between MetS and E-selectin and the gender-specific differences in the levels of inflammatory and endothelial dysfunction markers among subjects with or without MetS, especially E-selectin. In this study, we didn’t record the prevalence of menses and measure the hormonal status. Our results in females may therefore be influenced by this selection bias about hormonal status.

## Conclusions

Increased E-selectin levels are significantly associated with increased risk of MetS in Taiwanese females, but not in Taiwanese males. Increased awareness of the elevated levels of MetS-associated biomarkers, such as E-selectin may help early detection and management of individuals who are at risk for having MetS or its associated cardiovascular morbidity, particularly in individuals who already exhibit some clustered components of MetS.

## Supporting information

S1 Checklist STROBE checklist. (DOC)

S1 File E-selectin minimal dataset. (XLSX)
